# Plant Products as Inhibitors of Coronavirus 3CL Protease

**DOI:** 10.3389/fphar.2021.583387

**Published:** 2021-03-09

**Authors:** Anirban Mandal, Ajeet Kumar Jha, Banasri Hazra

**Affiliations:** ^1^ Department of Microbiology, Mrinalini Datta Mahavidyapith, Kolkata, India; ^2^ Animal Health Research Division, Nepal Agricultural Research Council, Kathmandu, Nepal; ^3^ Department of Pharmaceutical Technology, Jadavpur University, Kolkata, India

**Keywords:** coronavirus disease 2019, 3-chymotrypsin-like cysteine protease, main protease, SARS-CoV-2, plant products, plant-derived 3CLpro inhibitors

## Abstract

**Background:** The ongoing COVID-19 pandemic has created an alarming situation due to extensive loss of human lives and economy, posing enormous threat to global health security. Till date, no antiviral drug or vaccine against SARS-CoV-2 has reached the market, although a number of clinical trials are under way. The viral 3-chymotrypsin-like cysteine protease (3CL^pro^), playing pivotal roles in coronavirus replication and polyprotein processing, is essential for its life cycle. In fact, 3CL^pro^ is already a proven drug discovery target for SARS- and MERS-CoVs. This underlines the importance of 3CL protease in the design of potent drugs against COVID-19.

**Methods:** We have collected one hundred twenty-seven relevant literatures to prepare the review article. PubMed, Google Scholar and other scientific search engines were used to collect the literature based on keywords, like “SARS-CoVs-3CL protease,” “medicinal plant and anti-SARS-CoVs-3CL protease” published during 2003–2020. However, earlier publications related to this topic are also cited for necessary illustration and discussion. Repetitive articles and non-English studies were excluded.

**Results:** From the literature search, we have enlisted medicinal plants reported to inhibit coronavirus 3CL protease. Some of the plants like *Isatis tinctoria* L. (syn. *Isatis indigotica* Fort.)*, Torreya nucifera* (L.) Siebold and Zucc., *Psoralea corylifolia* L., and *Rheum palmatum* L. have exhibited strong anti-3CL^pro^ activity. We have also discussed about the phytochemicals with encouraging antiviral activity, such as, bavachinin, psoralidin, betulinic acid, curcumin and hinokinin, isolated from traditional medicinal plants.

**Conclusion:** Currently, searching for a plant-derived novel drug with better therapeutic index is highly desirable due to lack of specific treatment for SARS-CoV-2. It is expected that in-depth evaluation of medicinally important plants would reveal new molecules with significant potential to inhibit coronavirus 3CL protease for development into approved antiviral drug against COVID-19 in future.

## Introduction

Recently, a novel coronavirus was discovered in patients suffering from respiratory ailment accompanied by fever, dry cough and tiredness. Other symptoms, like sore throat, nasal congestion, headache, conjunctivitis, diarrhea, loss of taste or smell, were usually mild, and appeared gradually. This was coronavirus disease 2019 (COVID-19), unknown to the world before its outbreak in December 2019 in Wuhan, China (Burki, 2020). The highly infectious virus started to spread rapidly among the population in many countries all over the world, and created a pandemic situation within a couple of months (Bedford et al., 2020). Reports have indicated that even asymptomatic people can transmit the virus, mainly through respiratory droplets that can cause human-to-human transmission. The extremely contagious novel coronavirus 2019 (nCoV-19) was responsible for about 1.76 million deaths and 79.67 million confirmed cases globally till date (WHO, 2020).

The major phenotype of COVID-19 is severe acute respiratory distress syndrome (ARDS), similar to that caused by SARS-CoV and MERS-CoV (Hirano and Murakami, 2020). At present, there is no specific treatment for COVID-19, while vaccines will take some more time to come to the market. Hence, clinical management of COVID-19 is currently limited to preventive and supportive treatments, and mostly designed to alleviate further complications and organ damage (Rodríguez-Morales et al., 2020). Clinical studies have shown promising results in patients using the protease inhibitor drug lopinavir in combination with ritonavir, commonly used to treat HIV (Lu, 2020). Also, hydroxychloroquine, an antimalarial drug, and remdesivir, a nucleoside analogue of SARS-CoVs, were used to treat COVID-19 patients (Lee and Hsueh, 2020; Wu et al., 2020).

SARS-CoV-2 encodes two proteases, a papain like protease (PL^Pro^), and a 3- chymotrypsin-like cysteine protease (3CL^Pro^) also known as viral main protease (M^pro^), for proteolytic processing during viral maturation. The PL^Pro^ of coronavirus cleaves at no less than two sites on the pp1a polyprotein, whereas 3CL^Pro^ has at least eleven inter-domain sites on the pp1a and pp1ab polyproteins (Krichel et al., 2020). The functional importance of this proteolytic enzyme in the viral life cycle makes 3CL^pro^ a promising target for drug development against SARS-CoV-2 and other coronaviruses. Zhang et al. have successfully crystallized the 3CL protease from SARS-CoV-2 (Zhang et al., 2020a). This protease contains several highly conserved substrate-binding sites within the active site of the enzyme. Therefore, this is an opportunity for designing a wide variety of inhibitors against coronavirus 3CL^pro^. It is also exciting that the structures of 3CL^pro^ in SARS-CoV-2 and SARS-CoV differ by only twelve amino acids with comparable ligand binding efficiency (Macchiagodena et al., 2020). This demonstrated the possibility that inhibitors of SARS-CoV-3CL^pro^ will be active against SARS-CoV-2-3CL^pro^, too. In fact, protease inhibitors have drastically reduced the mortality against HCV/HIV, and maximized the therapeutic benefit (Kurt Yilmaz et al., 2016). Altogether, it is speculated that 3CL protease represents a potential target for the inhibition of CoV replication and this will definitely escalate the ongoing search for a new drug against SARS-CoV-2.

Natural products and their derivatives are used for treatment of various ailments since pre-historic times. A large number of herbal products and their constituents have shown promising inhibitory activity related to viral infections in humans (Ganta et al., 2017; Panda et al., 2017; Shen et al., 2019; Bahramsoltani and Rahimi, 2020). In 2002, during SARS-CoV outbreak in China, several clinical research projects were initiated on administration of Traditional Chinese Medicine (TCM) in combination with Western medicine for treatment of SARS-CoV infection (WHO, 2004). On verification of the clinical data, WHO declared that judicious use of TCM against SARS-CoV would help to reduce the mortality rate as compared to Western medicine alone. Furthermore, the combination therapy could lower the overall cost, and highlighted the importance of introducing plant products for the treatment of SARS-CoV (WHO, 2004).

**FIGURE 1:**
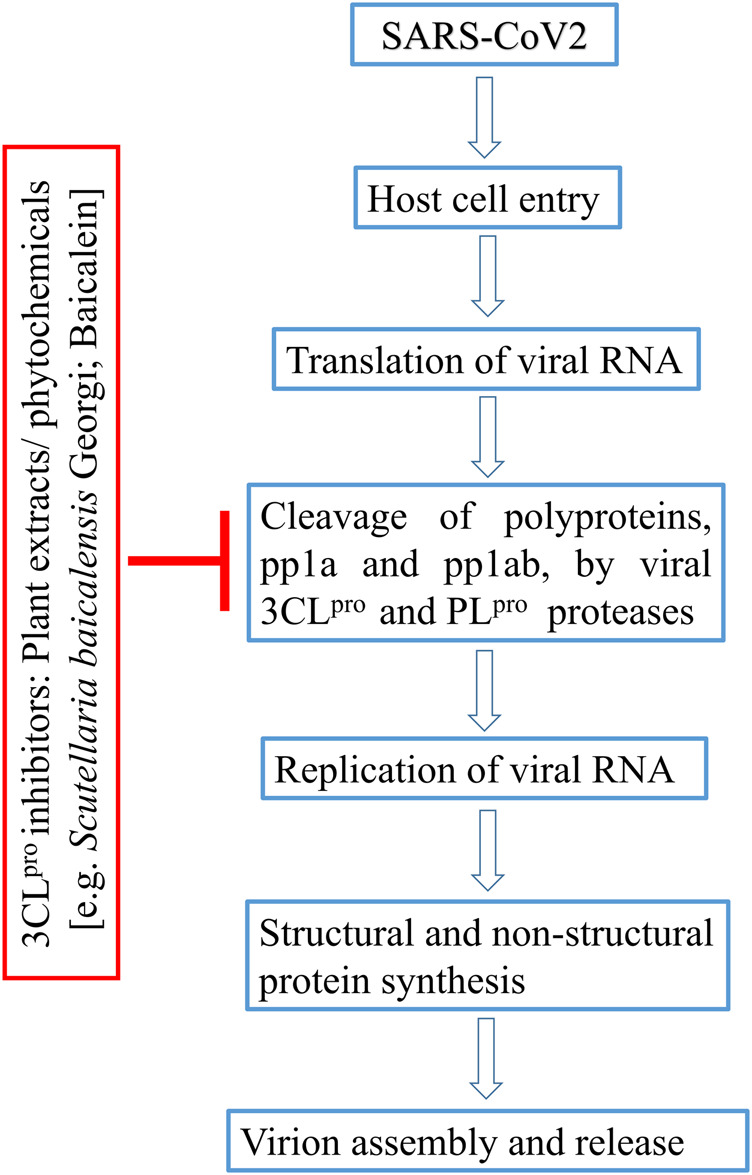
Flow-diagram on plant-derived inhibitors blocking 3CL^pro^ enzyme in SARS-CoV life cycle.

In this background, it would be relevant to search for plant products with potential efficacy to inhibit coronavirus 3CL protease (Figure 1). In fact, SARS-CoV-3CL^Pro^ was reportedly inhibited by phytochemicals abundant in green tea extracts (Chen et al., 2005). The therapeutic importance of green tea is evident in customary Indian and Chinese medicinal systems for prevention of various diseases (Haqqi et al., 1999; Kavanagh et al., 2001; Sueoka et al., 2001), and also for its, neuroprotective (Weinreb et al., 2004), anti-bacterial (Roccaro et al., 2004), and antiviral (Weber et al., 2003) properties. Flavanols and flavonols, including (-)-epigallocatechin-3-gallate (EGCG), are mainly responsible for health promoting effects of green tea (Higdon and Frei, 2003; Moyers and Kumar, 2004). Another traditional plant, *Angelica keiskei* (Miq.) Koidz (Umbelliferae), commonly used as a mild cathartic and diuretic tonic (Kimura and Baba, 2003), showed inhibitory activity against SARS-CoV-3CLpro with IC50 values of 11.4 µM (Park et al., 2016). This plant extract was also reported for its anti-bacterial, hepato-protective and other activities (Kil et al., 2017). *Cullen corylifolium* (L.) Medik. (syn. *Psoralea corylifolia* L.) is used in Chinese medicine and traditional Ayurveda against different types of skin diseases, such as leprosy, leukoderma and psoriasis (Sah et al., 2006). This plant is also known for its anti-inflammatory and antimicrobial properties (Khushboo et al., 2010). Later on, six aromatic compounds isolated from seeds of *Cullen corylifolium* (L)., Medik. were noted for their inhibitory activity against PL^pro^ (Kim et al., 2014); the isolated phytochemicals inhibited the enzyme in a dose-dependent manner with IC_50_ ranging from 4.2 to 38.4 µM. Similarly, many natural products have shown antiviral activity at nanomolar concentration against SARS-CoV (e.g., lycorine, homoharringtonine, silvestrol, ouabain, tylophorine and 7-methoxycryptopleurine), and might contribute to future drug discovery pipeline (Islam et al., 2020). In fact, clinical trials of a few herbal compounds against SARS-CoV-2-3CL^Pro^ aroused hope for plant-derived anti-SARS-CoV-2 drugs. Very recently, 3CL protease inhibitor NLC-001, a plant product administered orally as dietary supplement, got US FDA approval. Presently, clinical studies of NLC-001 to treat COVID-19 is going on in Israel while the pharmaceutical company Todos Medical Ltd. is evaluating its commercialization options worldwide (Golodetz, 2020). In another study, a natural product, baicalein, derived from TCM was identified as inhibitor of SARS-CoV-2-3CL^pro^ (Figure 1). Clinical trial showed that baicalein was well tolerated in treatment of acute, or chronic hepatitis in China (clinical trials registration number CTR20132944) (Li et al., 2014b).

In this review, we provided a compilation of well-documented plant-derived compounds and their derivatives acting against coronavirus 3CL protease, and their current stage of investigation. A detailed insight into the active compounds with regard to their mode of inhibition have been presented along with predictive studies *in silico*. Finally, a discussion is focused on several plants with ethno-pharmacological reputation. Further, we have proposed a few selected phytochemicals for evaluation against this protease enzyme with a view to providing clues toward coronavirus drug discovery in future.

## Methodology

**FIGURE 2:**
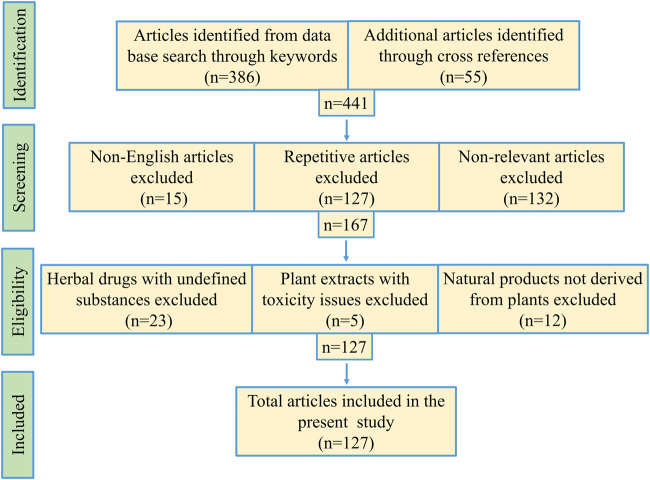
Schematic representation of methodology used for this study.

Scientific search engines PubMed, Google Scholar, ScienceDirect, SpringerLink, Scopus, EMBASE, Medline were critically investigated to obtain research articles with the help of search strings, viz. “SARS-CoVs-3CL protease,” “anti-SARS-CoV-2 drugs,” “medicinal plant and anti-SARS-CoVs-3CL protease,” “phytochemicals against SARS-CoVs-3CL protease,” “medicinal plants and HIV/HCV proteases,” etc. Research article inclusion criteria: 1) crude plant extracts, fractions or semi-purified fraction inhibiting coronaviruses, 2) isolated phytochemicals or its derivatives effective against coronaviruses, and 3) studies with medicinal plants acting against HIV/HCV proteases. Exclusion criteria: 1) literature duplication, 2) non-relevant articles, 3) studies involving herbal mixtures with undefined contents, 4) plant extracts with toxicity issues, 5) natural products not derived from plants. Total one hundred twenty-seven publications from 1994 to 2020 were cited in the present article (Figure 2). Mendeley desktop software was used for preparing the bibliography.

## Coronavirus Life Cycle

**FIGURE 3:**
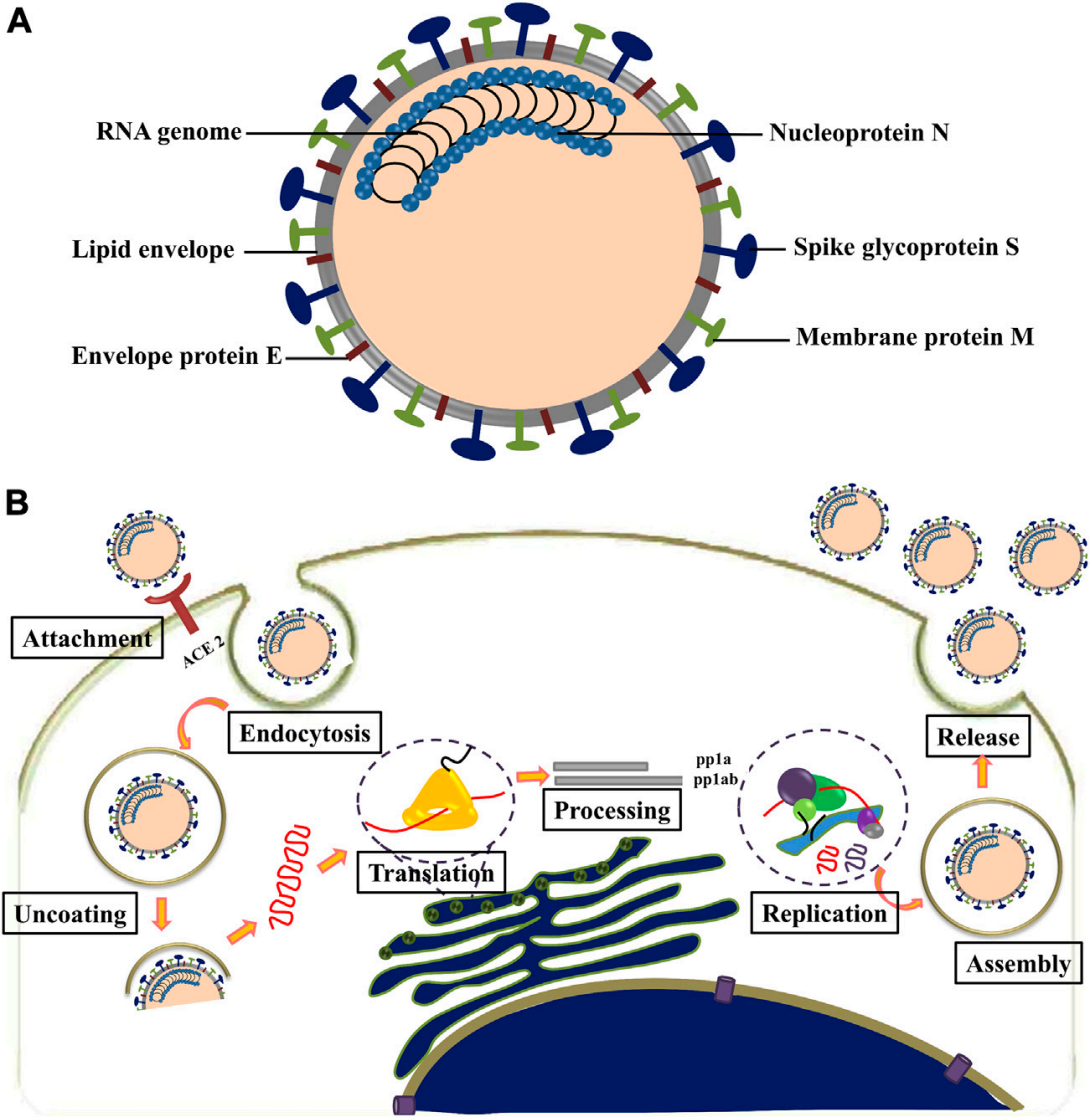
**(A)** Structure of coronavirus. **(B)** Life cycle of coronavirus.

SARS-CoV-2 is an enveloped, non-segmented, positive-sense RNA virus with genome size of ∼30 kb, belonging to the *Nidovirales* order and *Coronaviridae* family (Khailany et al., 2020). The viral genome contains at least six open reading frames (ORFs). The first ORF (ORF1a/b) is about two-thirds of the whole genome length and encodes 16 nonstructural proteins (nsp1-16). The virus contains four major structural proteins: spike (S), membrane (M), envelope (E), and nucleocapsid (N), all located within the 3′ end of the viral genome. Spike protein is responsible for binding with cellular receptors as well as fusion of viral and cellular membrane. M protein plays a central role in the assembly of viruses, making cellular membranes as workshops where viruses and host factors come together to create new virus particles. E protein is necessary for viral pathogenesis and its smaller size (9–12 kDa) makes it easier for the virus to assemble before release (Bianchi et al., 2020). N protein is the only protein in nucleocapsid that binds to the viral genome in a conformation of the “beads-on-a-string” type (Figure 3A) (Gordon et al., 2020). SARS-CoV-2 was found with ∼80% nucleotide identity to SARS coronavirus (Kim et al., 2020).

The attachment of SARS-CoV-2 to the host cell is initiated by interaction between S protein and its host cell receptor known as angiotensin-converting enzyme (ACE2) (Figure 3B) (Choudhary et al., 2020). This interaction is followed by a series of events leading to the delivery of viral genome into the cytoplasm. The 5′ end of the RNA genome, ORFs 1a and 1b, are translated into pp1a and pp1ab; upon entry into the cell pp1ab is translated through a frameshift mechanism. The non-structural proteins (nsps) 1–11 and 1–16 are encoded by polyproteins pp1a and pp1ab, respectively. These polyproteins are then cleaved into individual non-structural proteins by two viral proteases, namely, PLP and 3CL^pro^/M^pro^. The PLP encoded in nsp3, cleaves between nsp1/2, nsp2/3 and nsp3/4, while 3CL^pro^/M^pro^ encoded by nsp5 is responsible for the remaining 11 cleavage events (Krichel et al., 2020). Most of the non-structural proteins in the replicase–transcriptase complex (RTC) contribute to establish an environment favorable for RNA synthesis (Wang et al., 2020).

The synthesis of viral RNA produces genomic as well as sub-genomic RNAs. After viral RNA translation, the structural proteins S, E, and M are moved through secretory pathway into the endoplasmic reticulum–golgi intermediate compartment. The viral genomes, encapsulated in the compartment membrane, interact with the structural proteins to form mature virions and get transported to the cell surface to be released by exocytosis (Shereen et al., 2020).

## Structure of SARS-COV-3CL^PRO^

**FIGURE 4:**
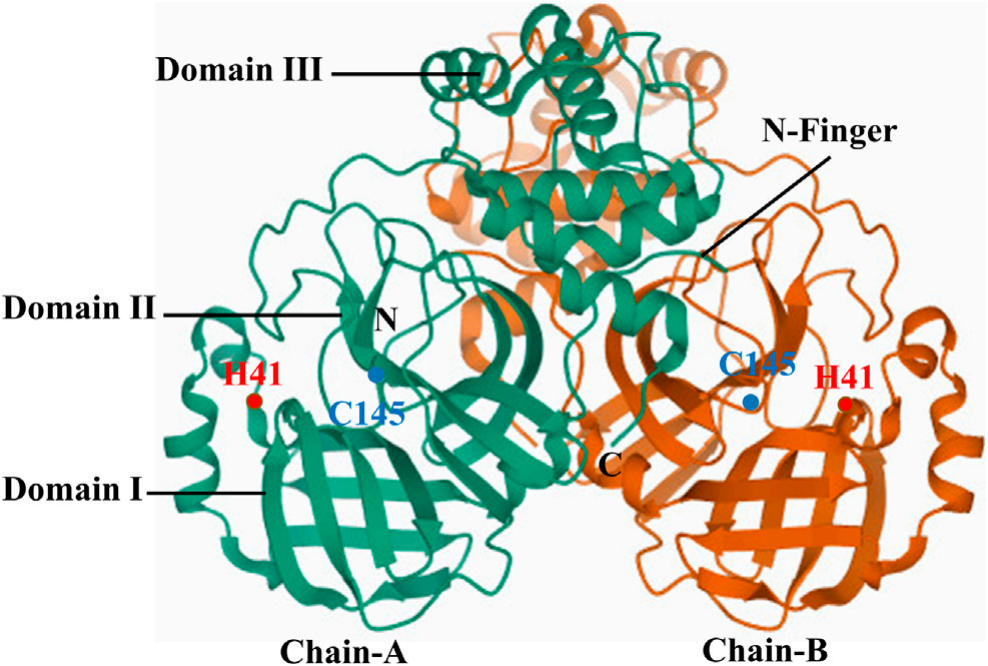
Crystal structure of SARS-CoV-2-3CL^pro^.

SARS-CoVs are single-stranded RNA viruses with the largest known viral RNA genomes to date. The genome is a polyadenylated RNA of ∼30 kb, and 41 percent of the residues are G or C rich (Rota et al., 2003). Functional polypeptides are released by extensive proteolytic processing from each polyprotein, primarily by 33.8-kDa 3C-like protease (Yang et al., 2003). The crystal structure of 3CL^pro^ (2.54 Å) reveals that the molecule is composed of three domains. Domains I, II and III contain amino acid residues 8 to 99, 100 to 183, and 184 to 303, respectively. Domains I and II are six-stranded antiparallel β barrels, and together mimic the architecture of chymotrypsin and 3C proteinases of picornavirus. Domain III is the substratum binding site found in a cleft between the two. A domain III loop connects domain II with C-terminal end of domain III (Anand et al., 2003). Domain III (residues 198–303), a globular cluster of five helices, regulates the dimerization of 3CL^pro^ through interaction of the salt-bridge between Glu290 of chain-A and Arg4 of chain-B (Shi and Song, 2006). The 3CL^pro^ active site contains a catalytic dyad in which a cysteine residue (Cys145) acts as a nucleophile, and histidine residue (His41) acts as a base (Figure 4) (Anand et al., 2003). SARS-CoV-2-3CL^pro^ has 96% structural similarity with SARS-CoV-3CL protease forming a dimeric structure for catalytic activity (Zhang et al., 2020a).

## Plants With Inhibitory Activity Against 3CL Protease

**FIGURE 5:**
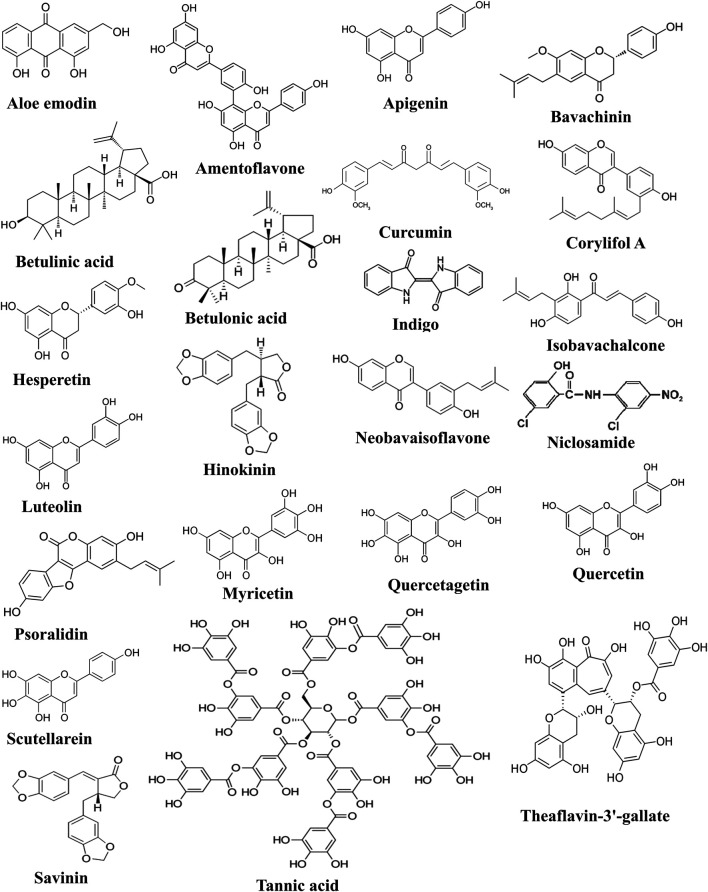
Chemical structures of compounds active against SARS-CoV-2-3CL^pro^.

Natural products have always played a crucial role in drug development against various diseases. Therefore, traditional herbs from diverse geographical locations and various habitats could be considered as potential sources of new drugs for treatment of viral infections, including those caused by SARS-CoVs and its emergent mutants. For centuries, the medicinal plant *Isatis tinctoria* L. (Brassicaceae) have been esteemed in Europe, Central Asia, and in TCM for therapeutic and cosmetic application, and indigo-blue dyeing character (Speranza et al., 2020). All parts of this plant are utilized in complementary and alternative medicinal preparations against eruptive epidemic diseases, pharyngitis, laryngitis, hepatitis, various kinds of fevers, influenza and viral skin diseases. Currently, *I. tinctoria* root is recognized in European phytotherapy for medicinal properties, mostly due to its antiviral activities (Hamburger, 2002). During the outbreaks of SARS-CoV in China, Hong Kong, and Taiwan, *Isatis tinctoria L.* and several phenolic herbs were commonly used against the viral disease. *I. tinctoria* L. root contains phytochemicals such as indigo, indirubin, indican, β-sitosterol, sinigrin and γ-sitosterol. Seven other compounds from various sources, namely aloe-emodin, hesperetin, quercetin, naringenin, daidzein, emodin and chrysophanol were also tested for their SARS-CoV-3CL^pro^ inhibitory effect (vide Table 1; Figure 5). Among these, aloe-emodin, sinigrin and hesperetin reportedly inhibited cleavage activity of the 3CL^pro^ in a cell-based assay in dose-dependent manner (Lin et al., 2005).

**TABLE 1:** Plant products with inhibitory activity against coronavirus 3CL protease.

Plant name [family]	Active principle	IC_50_ value	References
*Aloe vera* (L.) Burm.f. [Asphodelaceae]	Aloe emodin	8.3 µM	Lin et al. (2005)
*Citrus japonica* Thunb. [Rutaceae]	Hesperetin	217 µM	
*Isatis tinctoria* L. [Brassicaceae]	Sinigrin	365 µM	
	Indigo	752 μM	
*Torreya nucifera* (L.) Siebold and Zucc. [Taxaceae]	Biflavone amentoflavone	88.3 μM	Bae et al. (2010)
	Apigenin	280.8 μM	
	Luteolin	20.2 μM	
	Quercetin	23.8 μM	
*Cullen corylifolium* (L.) Medik. [Leguminosae]	Bavachinin	38.4 ± 2.4 μM	Kim et al. (2014)
	Corylifo	32.3 ± 3.2 μM	
	Isobavachalcone	18.3 ± 1.1 μM	
	4′-O-methylbavachalcone	10.1 ± 1.2 μM	
	Neobavaisoflavone	18.3 ± 1.1 μM	
	Psoralidin	4.2 ± 1.0 μM	
*Feretia apodanthera* subsp. *apodanthera* [Rubiaceae]	Betulinic acid	10 μM	Wen et al. (2007)
*Betula pendula* Roth [Betulaceae]	Betulonic acid	>100 μM	
*Curcuma longa* L. [Zingiberaceae]	Curcumin	40 μM	
*Phyllanthus niruri* L. [Phyllanthaceae]	Hinokinin	>100 μM	
*Pterocarpus santalinus* L.f. [Fabaceae]	Savinin	25 μM	
*Camellia sinensis* (L.) Kuntze [Theaceae]	Theaflavin-3′-gallate (black tea)	7 μM	Chen et al. (2005)
	Tannic acid (black tea)	3 μM	
	Theaflavin-3,3′-digallate (black tea)	9.5 μM	
*Scutellaria baicalensis* Georgi [Lamiaceae]	Baicalein	0.39 µM	Liu et al. (2020)
	Scutellarein	5.80 ± 0.22 µM	
	Dihydromyricetin	1.20 ± 0.09 µM	
	Quercetagetin	1.27 ± 0.15 µM	
	Myricetin	2.74 ± 0.31 µM	
*Rheum palmatum* L. [Polygonaceae]	RH10 fraction	38.09 ± 1.70 (μg/mL)	Luo et al. (2009)
	RH11 fraction	22.30 ± 1.26 (μg/mL)	
	RH12 fraction	59.33 ± 6.52 (μg/mL)	
	RH121 fraction	13.76 ± 0.03 (μg/mL)	
	RH122 fraction	34.01 ± 5.68 (μg/mL)	
	RH124 fraction	52.43 ± 4.52 (μg/mL)	
	RH125 fraction	20.53 ± 3.20 (μg/mL)	

*Torreya nucifera* (L.) Siebold and Zucc. is historically used as a medicinal herb in Asia. Ethanol extract of *T. nucifera* leaves showed promising inhibitory activity against SARS-CoV-3CL^pro^ (62% viral inhibition at 100 μg/ml). Eight diterpenoids and four biflavonoids were isolated and evaluated for SARS-CoV-3CL^pro^ inhibition using fluorescence resonance energy transfer analysis following bioactivity-guided fractionation. Out of these compounds, the most potent inhibitory effect on 3CL^pro^ was shown by a biflavone, namely, amentoflavone (IC_50_ = 8.3 µM). Three more biflavones, viz. apigenin, luteolin and quercetin inhibited 3CL^pro^ activity with IC_50_ values of 280.8, 20.2, and 23.8 µM, respectively (Bae et al., 2010).

Ethanolic extract of *Cullen corylifolium* (L.) Medik (syn. *Psoralea corylifolia* L.) seeds showed high activity against the SARS-CoV-PL^pro^ with an IC_50_ value of 15 μg/ml. Subsequently, bioactivity-guided fractionation of the ethanol extract resulted in six aromatic compounds known as bavachinin, neobavaisoflavone, isobavachalcone, 4′-O-methylbavachalcone, psoralidin and corylifol A. All the isolated flavonoids inhibited PL^pro^ to exhibit their inhibitory potency in a dose-dependent manner (Kim et al., 2014).

Another study with twenty phytocompounds, including abietane and labdane-type diterpenes, lupane-type triterpenes, lignoids and curcumin, were tested for their anti-SARS-CoVs activity. The tested phytocompounds exhibited substantial levels of anti-SARS-CoV activity at 10 μM. Betulinic acid, betulonic acid, hinokinin, curcumin, niclosamide and savinin were among the twenty phytocompounds showing substantial inhibition of 3CL protease. It was claimed as the first report to demonstrate that natural lupane-type triterpenes and lignan could block 3CL protease activity by competitive inhibition (Wen et al., 2007).

Various types of tea extracts including black tea, green tea, oolong tea, and pu'er tea were investigated for 3CL^Pro^ inhibitory activity. Results suggested that extracts from pu'er and black tea were more potent than those from green or oolong tea. Finally, water extracts from different types of tea were prepared and tested for their inhibitory activities against 3CL^Pro^ (Chen et al., 2005). The authors found that 3CL^Pro^ inhibitors were tannic acid, theaflavin-3′-gallate and theaflavin-3, 3′-digallate, as revealed by the fluorogenic substrate assay.

The inhibitory effect of sixty-four purified natural compounds on the function of SARS-CoV helicase, nsP13, and HCV helicase was determined by double-strand DNA unwinding assay, or fluorescence resonance energy transfer, or colorimetry-based ATP hydrolysis assay. The study showed that a few selected natural flavonoids, including myricetin and scutellarein, may serve as potent inhibitors of SARS-CoV (Yu et al., 2012). Recently, traditional Chinese medicinal plant *Scutellaria baicalensis* Georgi, widely used as broad spectrum antiviral agent, demonstrated anti-SARS-CoV-2-3CL^pro^ activity *in vitro* with EC_50_ value 0.74 μg/ml (Liu et al., 2020). The active molecule baicalein strongly inhibited the enzymatic activity with IC_50_ value 0.39 µM. Again, the authors identified four baicalein analogues, namely, scutellarein, dihydromyricetin, quercetagetin, and myricetin could inhibit SARS-CoV-2-3CL^pro^ activity at micromolar concentration.

The alcoholic extract of Chinese medicinal plant *Rheum palmatum* L. roots and rhizomes were studied against the protease enzyme; the semi-purified fractions such as RH11, RH121, and RH125 significantly inhibited 3CL protease activity of SARS coronavirus (Luo et al., 2009).

## In Silico Studies on Phytochemicals Against 3CL-Protease

Phytochemical screening *in silico* is particularly appealing because it can virtually screen thousands of compounds within a stipulated time, and scrutinize the prospect of drug-like molecules. Several *in silico* studies revealed potential anti-SARS-CoV-3CL^pro^ natural compounds using molecular docking studies. Induced-fit docking analysis showed that three flavonoids, namely herbacetin, rhoifolin and pectolinarin could block the enzymatic activity of SARS-CoV-3CL^pro^ and suggested as templates for designing functionally improved inhibitors (Jo et al., 2020). Another recent study by Tahir ul Qamar et al. (2020) identified nine potential SARS-CoV-3CL^pro^ phytochemicals, namely; 5,7,3′,4′-tetrahydroxy-2′-(3,3-dimethylallyl) isoflavone, myricitrin, calceolarioside B, methyl rosmarinate, 3,5,7,3′,4′,5′-hexahydroxy flavanone-3-O-beta-D-glucopyranoside, (2S)-eriodictyol 7-O-(6′-O-galloyl)-beta-D-glucopyranoside, myricetin 3-O-beta-D-glucopyranoside, licoleafol, and amaranthin after screening of a medicinal plant database containing 32,297 potential antiviral phytochemicals (Tahir ul Qamar et al., 2020). Another *in silico* study demonstrated andrographolide as a potential inhibitor of coronavirus main protease, with good pharmacodynamic property and target accuracy (Enmozhi et al., 2020). Molecular docking study revealed that several flavonoids from *Salvadora persica* L. inhibited SARS-COV-2-3CL protease (Owis et al., 2020). Previously, the proteolytic activity of SARS-CoV-3CL^pro^ was found to be inhibited by apigenin, luteolin, quercetin, amentoflavone, daidzein, puerarin, epigallocatechin, epigallocatechin gallate, gallocatechin gallate and kaempferol (Bae et al., 2010; Thanh et al., 2012; Efferth and Schwarz, 2014). Similarly, several natural alkaloids and terpenoids could inhibit 3CL^pro^ of both SARS-CoV-2 and SARS-CoV with highly conserved inhibitory pattern. In another study, some African plants were screened using *in silico* approach to derive alkaloids and terpenoids as potential inhibitors of coronavirus 3CL^pro^ (Gyebi et al., 2020). Twenty alkaloids and terpenoids with high binding affinities to SARS-CoV-2-3CL^pro^ were additionally docked in SARS-CoV and MERS-CoV-3CL^pro^. A strongly specified hit-list of seven compounds (10-hydroxyusambarensine, cryptoquindoline, 6-oxoisoiguesterine, 2-hydroxyhopan-3-one, cryptospirolepine, isoiguesterine, and 20-epibryonolic acid) were identified in the ligand-protein interaction analysis (Gyebi et al., 2020). In order to explore the antiviral activity of well-known phytochemicals, like kaempferol, quercetin, luteolin-7-glucoside, demethoxycurcumin, naringenin, apigenin-7-glucoside, oleuropein, curcumin, catechin, and epicatechin-gallate against COVID-19 M^pro^ protein, a molecular docking study was recruited, which strongly correlated the inhibitory activity of the selected phytochemicals (Khaerunnisa et al., 2020). A docking study was performed to find one hundred eighteen constituents, with high binding affinity toward SARS-CoV-2-3CL^pro^, identified from Respiratory Detox Shot, a TCM prescription for COVID-19 control and prevention. Subsequently, *in vitro* assessment performed on the drug like candidates using the 3CL^pro^ inhibition assay could validate twenty-two active constituents (Zhang et al., 2020c).

## Future Prospect

### Promising Plants and Related Natural Products Waiting to be Evaluated against SARS-CoV-2-3CL^pro^

**FIGURE 6:**
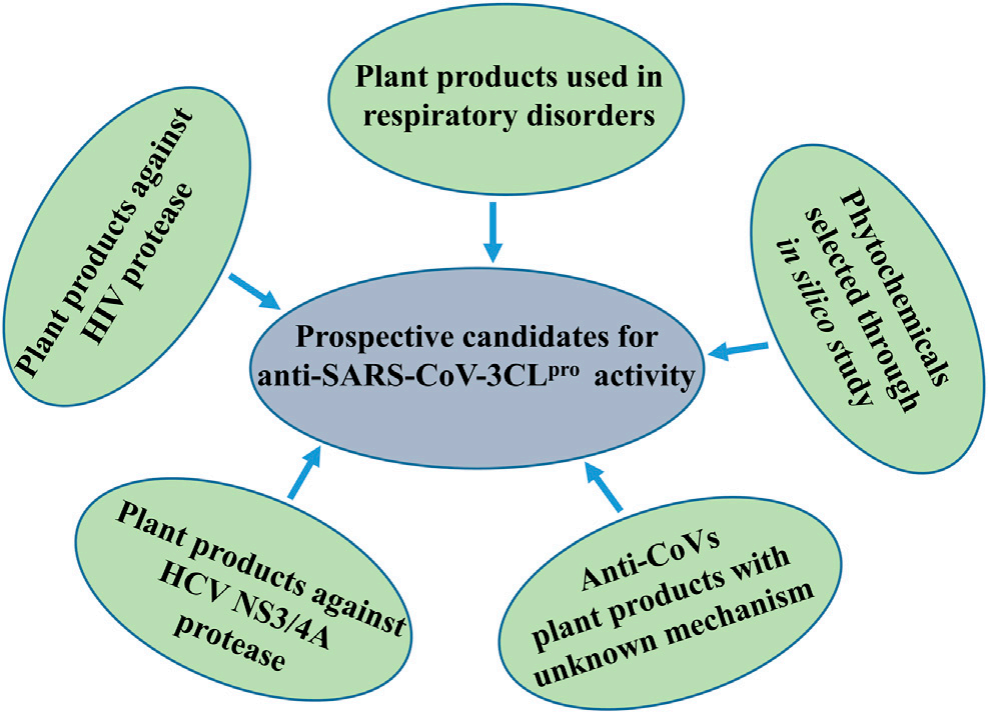
Schematic demonstration of prospective candidates for anti-SARS-CoV-3CL^pro^ activity.

Considering the extensive global research focusing on antiviral plant products, the prospect of discovering anti-SARS-CoV-2 drugs remains hopeful. In fact, several bioactive constituents derived from traditional medicinal plants have now gained much attention as prophylactic immunity-boosters and/or adjuvant therapy for management of SARS-CoV-2. In the earlier sections, we have presented a number of phytochemicals and plant products inhibiting coronavirus 3CL protease. Now we shall enlist some of the prospective medicinal plants waiting for evaluation against coronavirus 3CL^pro^, and present them in Tables 2–6. In future, it would be more meaningful if advanced technology is used for cross-screening of these plants, as shown in Figure 6, for their potential activity against SARS-CoV-2.

**TABLE 2:** HIV protease inhibiting plants waiting to be evaluated against SARS-CoV-2-3CL^pro^.

Name and family	Active part	Extract type/Molecule	EC_50_ (μg/ml)	CC_50_ (μg/ml)	References
*Alisma plantago-aquatica* subsp. *orientale* (Sam.) Sam. [Alismataceae]	Rh	Hot water	29.2	394.2	Xu et al. (1996)
*Andrographis paniculata* (Burm. f.) Nees [Acanthaceae]	WP	Water	7.2	210	Otake et al. (1995)
*Camellia japonica* L. [Theaceae]	Pc	Camelliatanin H	90	≥166.6	Park et al. (2002)
*Crotalaria pallida* Aiton [Fabaceae]	S, F	Methanol, Ethanol	23	127.0	Govindappa et al. (2011)
*Erythroxylum citrifolium* A.St.-Hil. [Erythroxylaceae]	Tr	Water	50	62	Govindappa et al. (2011)
*Geum japonicum* Thunb. [Rosaceae]	WP	Maslinic acid	ND	ND	Matsuse et al. (1998)
*Helichrysum populifolium* DC. [Asteraceae]	L, Ss	Ethanol	ND	ND	Xu et al. (1996)
*Justicia adhatoda* L. [Acanthaceae]	L	Water	6.7 ± 5.2	≤1,000	Singh et al. (2010)
*Mangifera indica* L. [Anacardiaceae]	L	Mangiferin	27.3 ± 2.3	≥500	Wan et al. (1996)
*Mentha villosa* Huds. [Lamiaceae]	L	Flavones/Menthalactone	1.8	32.6	Hinay and Sarol (2014)
*Plectranthus amboinicus* (Lour.) Spreng. [Lamiaceae]	L	Ethanol	123.7	726.2 ± 3.1	Thayil et al. (2016)
*Rhus chinensis Mill.* [Anacardiaceae]	Bb, G	Hot extract	ND	ND	Xu et al. (1996)
*Waltheria indica* L. [Malvaceae]	Br	Water	48	154.2	Matsuse et al. (1998)
*Xylopia frutescens* Aubl. [Annonaceae]	B	Methanol	46	56.6	Matsuse et al. (1998)

AP, erial part; B, bark; Bb, bulb; Br, branch; F, fruit; G, gall; L, leaf; ND, not determined; Pc, pericarp; Rh, rhizome; S, stem; Ss, seeds; Tr, trunk; WP, whole plant.

**TABLE 3:** HCV protease inhibiting plants waiting to be evaluated against SARS-CoV-2-3CL^pro^.

Plant name (family)	Active part	Extract type/Molecule	% of inhibition	IC_50_ (μg/ml)	References
*Boswellia carterii* Birdw. [Burseraceae]	R	Methanol	100.0 ± 0.0	23.0	Hussein et al. (2000)
		Water	71.6 ± 2.5	ND	
*Embelia ribes* Burm [Myrsinaceae]	F	Methanol	93.9 ± 0.4	38.0	Bachmetov et al. (2012)
		Embelin	96.6 ± 0.9	21	
		5-O-Methylembelin	77.9 ± 1.7	46	
*Phyllanthus amarus* Schumach. and Thonn. [Phyllanthaceae]	R	Methanol	ND	ND	Ravikumar et al. (2011)
*Punica granatum* L. [Punicaceae]	FP	Methanol	89.9 ± 0.9	ND	Hussein et al. (2000)
		Water	84.9 ± 1.8	ND	
*Trichilia emetica* Vahl [Meliaceae]	B	Methanol	57.3 ± 3.7	ND	Hussein et al. (2000)
*Vachellia nilotica (L.)* P.J.H. Hurter and Mabb. [Fabaceae]	B	Methanol	91.0 ± 0.0	ND	Hussein et al. (2000)
*Vitis vinifera* L. [Vitaceae]	R	Vitisin B	3.0	>10.0	Lee et al. (2016)

B, Bark; F, Fruit; FP, Fruit Pericarp; ND, not determined; R, root.

**TABLE 4:** Medicinal plants, used in respiratory disorders, with potential activity against SARS-CoV-2-3CL^pro^.

Name and family	Active part	Traditional use	Pharmacological effect	References
*Broussonetia papyrifera* (L.) L’Hér. ex Vent. [Moraceae]	F	Cough	Protection of bronchitis and inflammation in lungs	Ko et al. (2013)
*Carum carvi* L. [Asteraceae]	S, L	Bronchitis, cough	Anti-cholinergic, bronchodilator	Boskabady and Shahabi (1997)
*Ficus religiosa* L. [Papilionaceae]	F, L	Asthma	Mast cell stabilizing effect in bronchospasm animal model	Kapoor et al. (2011)
*Hyoscyamus niger* Linn. [Zygophyllaceae]	WP	Asthma, Whooping cough	Bronchodilatory effect through blockade of Ca^2+^ channels and muscarinic receptors	Gilani et al. (2008)
*Mimosa pudica* Linn. [Lamiaceae]	R, L	Asthma	Bronchodilatory effect in bronchospasm animal model	Mail et al. (2011)
*Salvia officinalis* Linn. [Liliaceae]	L	Cough, cold	Bronchodilatory effect through activation of K^+^ channels and phosphodiesterase inhibition	Gilani et al. (2007)
*Spinacia oleracea* L. [Amarylliadaceae]	L	Cough	Anti-asthmatic effect in animal model	Yamada et al. (2010)
*Vitex negundo* Linn. [Verbenaceae]	L	Flu	Anti-inflammatory, anti-asthmatic, anti-allergic and bronchodilatory activity	Patel et al. (2011)

F, fruit; L, leaf; R, root; S, stem; WP, whole plant.

**TABLE 5:** Phytochemicals selected for anti-SARS-CoV-2-3CL^pro^ activity i*n silico*.

Plant name (family)	Active compound	Binding energy	References
*Allium fistulosum* L. [Amaryllidaceae]	Luteolin-7-glucoside	−8.17	Khaerunnisa et al. (2020)
*Allium sativum* L. [Amaryllidaceae]	Allicin	−4.03	Khaerunnisa et al. (2020)
*Andrographis paniculata* (Burm. f.) Nees [Acanthaceae]	Andrographolide	−3.09	Enmozhi et al. (2020)
*Anethum graveolens* L. [Umbellifers]	Kaempferol	−8.58	Khaerunnisa et al. (2020)
*Averrhoa bilimbi* L. (Juss.) M.Roem. [Oxalidaceae]	Apigenin-7-glucoside	−7.83	Khaerunnisa et al. (2020)
*Citrus aurantium* L. [Rutaceae]	Naringenin	−7.89	Khaerunnisa et al. (2020)
*Curcuma longa* L. [Zingiberaceae]	Demethoxycurcumin	−7.99	Khaerunnisa et al. (2020)

**TABLE 6:** SARS-CoV inhibiting plants waiting for *in vitro* anti-SARS-CoV-2-3CL^pro^ activity.

Plant name (family)	Active part	Extract type/Molecule	EC_50_	CC_50_ (μg/ml)	References
*Capsella bursa-pastoris* (L.) Medik [Brassicaceae]	WP	Butanol	43.1 ± 2.8 μg/ml	283.4 ± 16.3 μg/ml	Zhuang et al. (2009)
*Houttuynia cordata* Thunb. [Saururaceae]	L	Ethyl acetate	0.98 μg/ml	NC	Chiow et al. (2016)
*Lycoris radiata* (L’Hér.) Herb. [Amaryllidaceae]	L	Lycorine	15.7 ± 1.2 nM	14,980 ± 912 nM	Li et al. (2005)
*Toona sinensis* (Juss.) M.Roem. [Meliaceae]	L	Water	30 μg/ml	>500 μg/ml	Chen et al. (2008)
*Veronica linariifolia* Pall. ex Link [Plantaginaceae]	WP	Luteolin	9.02 μM	155 μM	Wu et al. (2004)
*Vitis vinifera* L. [Vitaceae]	Sk	Resveratrol	125–250 μM	>250 μM	Lin et al. (2017)

NC, no cytotoxicity at all tested concentrations; L, leaf; R, root; Sk, skin; WP, whole plant.

It has been reported that HIV protease inhibitors could be used against coronavirus by targeting SARS-CoV-2-3CL^pro^. To deal with the COVID-19 pandemic, HIV protease inhibitors (lopinavir/ritonavir) are currently being used with some success. Both the drugs are active against SARS-CoV-2-3CL^pro^ (Chen et al., 2020). Hence, we searched for the plants which have already demonstrated inhibitory activity against HIV protease, for example, *Justicia adhatoda* L (Acanthaceae) leaf extract and *Andrographis paniculata* (Burm. f.) Nees listed in Table 2. Therefore, these plants should be specifically investigated for the probability of anti-SARS-CoV-2-3CL^pro^ activity as well. Again, mangiferin, isolated from *Mangifera indica* L., demonstrated efficacy against mutant strains of HIV-1 protease. Hence, mangiferin should be tasted against resistance strains of SARS-CoV-2-3CL^pro^. Flavones/menthalactone isolated from *Mentha villosa* Huds. inhibited HIV-protease at post-translational level, therefore, may also inhibit polyprotein processing activity of SARS-CoV-2-3CL^pro^. Similarly, natural products like camelliatanin H and maslinic acid could be tested for application against coronavirus.

Structural similarity of HCV NS3/4A protease and SARS-CoV-2-3CL^pro^ suggested a promising approach for finding useful plant products for COVID-19 therapy (Bafna et al., 2020). Both the proteases have a striking 3D-structural similarity, particularly in the key active site residues. Hence, plants which are already known to possess inhibitory activity against HCV NS3/4A protease need to be tested against coronavirus also (Table 3). Thus, plants like *Vachellia nilotica* (L.) P.J.H.Hurter and Mabb. (syn. *Acacia nilotica* (L.). Willd. ex Delile), *Boswellia carterii* Birdw., *Embelia ribes* Burm. f., *Phyllanthus amarus* Schumach. and Thonn. should be repurposed as coronavirus therapeutics.

Following the outbreak of SARS-CoV in 2002, research efforts to find new antiviral agents were rationally focused on traditional medicinal plants reputed in different parts of the world for treatment of pulmonary disorder, asthma and fevers related to cough and cold problems and bronchial infections. The plants enlisted in Table 4 have been commonly used as anti-allergic/anti-histamine/bronchodilator/muscle relaxant in folk cultures, and well-known for immunomodulatory, anti-inflammatory and antioxidant properties. All of these features are useful for the treatment of respiratory disorders (Savithramma et al., 2007). Therefore, these medicinal plants should be evaluated for anti-SARS-CoV-2 activity, since respiratory disorder is an expected outcome of COVID-19 infection. For example, *Broussonetia papyrifera* L., and *Artemisia scoparia* Waldst. and Kit. are traditionally used for common cough, lung inflammation, bronchitis and asthma. Medicinal plants such as *Salvia officinalis* Linn. and *Carum carvi* L. pharmacologically act as bronchodilators, while *Ficus religiosa* L, *Mimosa pudica* Linn. and *Hyoscyamus niger* Linn. are used for anti-asthmatic purposes. Some such plants, enlisted in Table 5, are waiting to be explored for unknown antiviral constituents, as well as for their prospective anti-SARS-CoV-2-3CL^pro^ activity.

Further, evidences from *in silico* molecular docking studies have suggested potential anti-SARS-CoV-3CL^pro^ activity of plants, like *Allium sativum* L., *Anethum graveolens* L., *Citrus aurantium* L., *Curcuma longa* L, *Zingiber officinale* Roscoe, as given in Table 5. Molecular docking study showed that the binding energy of SARS-CoV-3CL^pro^ with luteolin-7-glucoside, allicin, andrographolide, kaempferol, apigenin-7-glucoside, naringenin, and demethoxycurcumin, were −8.17, −4.03, −3.09, −8.58, −7.83, −7.89, and −7.99 kcal/mol, respectively. The docking analysis indicated the comparative inhibition potential of these compounds in the following order: kaempferol > luteolin-7-glucoside > demethoxycurcumin > naringenin > apigenin-7-glucoside > allicin > andrographolide. Hence, these phytochemical constituents of medicinal plants could be explored as potential inhibitors of SARS-CoV-3CL^pro^.

Again, the specific mechanisms of antiviral action of certain plants, as shown in Table 6, are either unknown, or by way of some other inhibitory pathway, subject to experimental validation through anti-SARS-CoV-3CL^pro^ activity. Butanol extract of *Capsella bursa-pastoris* (L.) Medik showed inhibitory activity against SARS-CoV with IC_50_ value of 283.4 ± 16.3 μg/ml, and EC_50_ = 43.1 ± 2.8 μg/ml. Chen et al. (2008) reported for the first time that leaf extract of Toona sinensis Roem. can inhibit SARS-CoV. Another report described superior antiviral efficacy of ethyl acetate extract isolated from *Houttuynia cordata* Thunb. Therefore, these plants would serve as important clues awaiting further exploration on their putative anti-SARS-CoV-3CL^pro^ activity.

Lycorine isolated from *Lycoris radiata* (L’Hér.) Herb. is an effective antiviral compound against coronavirus. Again, resveratrol (terpenoid) and luteolin (flavonoid) extracted from *Veronica linariifolia* Pall. ex Link strikingly inhibited coronavirus entry by interfering with the binding between ACE2 receptor and spike protein of SARS-CoV. These compounds have antiviral potential against coronavirus and need experimental validation in order to confirm their anti-SARS-CoV-3CL^pro^ activity.

### Selection of Phytochemicals Suitable for Future Development Against SARS-CoV-3CL^pro^

Following the outbreak of SARS-CoV in 2002, research efforts were focused on the antiviral prospect of selected constituents from medicinal plants which are traditionally used for community health care in different parts of the world. Some of them are presented below with relevance to their development against COVID-19, in future.

#### Betulin Analogues

Betulin and betulinic acid (pentacyclic lupane-type triterpenes) are ubiquitous secondary metabolites found in a variety of medicinal plants, mainly of Betulaceae family, possessing a wide range of pharmacological activities. The bark extracts of several species of birch trees (*Betula spp*.) have been recommended in traditional medicinal systems for the treatment of rheumatism and arthritis, hepatitis, skin rash, intestinal worms and scurvy (Rastogi et al., 2015). Plenty of investigations have been undertaken to clarify the ethnopharmacological aspects, which showed that betulinic acid and its analogues could be useful for the treatment of cancer, inflammatory diseases, metabolic disorders, cardiovascular conditions, and neurological ailments (Ebeling et al., 2014; Amiri et al., 2020). Moreover, betulin and betulinic acid were shown to be non-toxic, with favourable therapeutic index at doses up to 500 mg/kg body weight in mice (Jäger et al., 2008). The promising antiviral activity of betulin and betulinic acid was reported by several workers (Fujioka et al., 1994; Pavlova et al., 2003). Further studies indicated that betulinic acid acts in the early phase of HIV infection by preventing cleavage of Gag protein, thereby hindering maturation of the virus (Bildziukevich et al., 2019). Again, Navid et al., 2014 demonstrated effective inhibition of herpes virus by analogues of betulin against acyclovir-resistant clinical isolates of HSV-1; also, hepatitis B virus has been found to be susceptible to betulin analogues (Yao et al., 2009; Navid et al., 2014). In a study by Wen et al. (2007), phyto-compounds were evaluated for antiviral activity against SARS-CoV (Wen et al., 2007). Out of 221 compounds, betulinic acid was found to be the most effective inhibitor of the coronavirus. Then, on the basis of molecular modeling analysis, it was observed that the competitive inhibitory activity of betulinic acid on 3CL^pro^ activity was consistent with the formation of multiple hydrogen bond interactions between the compound and specific amino acid residues located at the active site of the pocket of the protease enzyme (Pillaiyar et al., 2016). Taken together, this indicated practical applicability of betulinic acid and its derivatives to provide a precise direction for development of novel anti-SARS-CoVs-3CL^pro^ drugs from natural sources.

#### Griffithsin

Griffithsin, an algal protein, was isolated from an aqueous extract of *Griffithsia* sp (Ceramiaceae genus) of marine red alga (Rhodophyta). Traditionally, red algae have a long history of use as functional foods for their richness in protein, fatty acids, minerals and vitamins (Li et al., 2014a). The potent anti-HIV activity of griffithsin was found with EC_50_ at a pico-molar range, and much lesser toxicity to the host cells (Mori et al., 2005). Later on, griffithsin was considered for topical application as a pre-exposure prophylactic against HIV (O’Keefe et al., 2009). In fact, it has been found that the lectin could inactivate other enveloped viruses also, especially those with highly glycosylated proteins on their surface (Kouokam et al., 2016). The protein showed inhibitory activity against a broad spectrum of animal and human coronaviruses, as it could bind specifically to the SARS-CoV spike glycoprotein and inhibited entry of the virus (O’Keefe et al., 2010). The outstanding efficacy of griffithsin in SARS-CoV-infected mice suggested that it merits further investigation for prophylaxis or treatment of respiratory infection caused by emerging viruses of the Coronaviridae family. Thus, by virtue of its broad antiviral spectrum, griffithsin holds great promise for development into universal antiviral therapeutics. Therefore, this molecule needs further exploration on its putative anti- SARS-CoV-3CL^pro^ activity.

#### Glycyrrhizin

Glycyrrhizin (or glycyrrhizic acid) is a major constituent of liquorice root obtained from *Glycyrrhiza glabra* L., and *Glycyrrhiza uralensis* Fisch. ex DC. from Fabaceae family of plants. Liquorice extract is a well-known phytomedicine in use since prehistoric times to alleviate common ailments like bronchitis, gastritis and jaundice. Currently, it is added as edible emulsifier and gel-forming agent in modern foodstuffs, and investigated for its anti-inflammatory, anti-allergenic, antimicrobial and antiviral properties in order to validate its traditional medicinal applications (Li et al., 2014a). Oriental medicinal systems recommended it as antitussive treatment of viral respiratory tract infections, such as dry cough or hoarse voice, and also for chronic fevers (Shibata, 2000). Actually, randomized controlled trials conducted with glycyrrhizin and its derivatives reduced hepatocellular damage in patients with HIV-1, and chronic hepatitis B and C virus infection. Additionally, animal studies on glycyrrhizin demonstrated a reduction of mortality in HSV, and influenza A virus (Fiore et al., 2008). *In vitro* studies revealed antiviral activity against HIV-1, SARS-CoV, respiratory syncytial virus, arboviruses, vaccinia virus and vesicular stomatitis virus (Baltina et al., 2015). Further studies on clinical isolates of coronavirus showed efficacy of glycyrrhizin to inhibit viral replication, as well as the adsorption and penetration of virus into host cell at non-cytotoxic concentration (Cinatl et al., 2003). Further, chemically modified glycyrrhizin derivatives exhibited enhanced inhibition of SARS-CoV replication *in vitro* (Hoever et al., 2005). Recent studies based on integrated computational approach and pharmacological aspects lend further support to glycyrrhizin playing an auxiliary role in COVID-19 treatment (Luo et al., 2020; Muhseen et al., 2020). In a timely review, Bailly and Vergoten (2020) have critically analyzed the prospective development of glycyrrhizin analogues not only as antiviral drugs, but also as adjuvant therapy for their protective effects on the vulnerable organs in patients suffering from SARS-CoV-2 infection (Bailly and Vergoten., 2020). Taking all this into consideration it is a high time to explore this time-tested safe phytochemical against SARS-CoV-2-3CL^pro^ to an approved drug in future.

#### Lycorine

Lycorine is a bioactive constituent of Amaryllidaceae family of plants, like *Hymenocallis littoralis* (Jacq.) Salisb., *Lycoris radiata* (L'Hér.) Herb., and *Narcissus pseudonarcissus* L. cv. Dutch Master, which are traditionally used in many countries for wound healing, and treatment of cancer and infectious diseases (Nair and Van Staden, 2014). In China, the bulbs of *Lycoris radiata* have been traditionally used in the treatment of laryngeal complications, wounds and carbuncles (He et al., 2013). Lycorine potently inhibited flaviviruses in cell culture mainly through suppression of viral RNA replication (Zou et al., 2009). In fact, this alkaloid has been found to exhibit a wide range of antiviral activities against ZIKA, HIV-1, HCV, and SARS-CoV (Liu et al., 2011). A high throughput screening study on two hundred herbal extracts demonstrated *Lycoris radiata* (L'Hér.) Herb. to be the most potent antiviral plant against SARS-CoV with EC_50_ = 2.4 ± 0.2 μg/ml. Further, meticulous fractionation and analysis of *L. radiata* extract led to the identification of lycorine as the active principle, with EC_50_ = 15.7 ± 1.2 nM (Li et al., 2005). In a recent study, the mechanism behind the anti-SARS-CoV-2 activity of lycorine has been attributed to modulating the host factors (Zhang et al., 2020b). Nevertheless, it would be interesting to explore this molecule as a candidate for anti-3CL^pro^ activity in future.

#### Tanshinone

Tanshinones are a class of phenanthrene-quinone diterpenoids that are the major lipophilic constituents of the root of *Salvia miltiorrhiza* Bunge, a well-known traditional Chinese medicinal herb (Danshen), primarily used for treating cardiovascular and cerebrovascular diseases. More than 40 types of tanshinone molecules have been characterized exhibiting a variety of biological activities in traditional clinical applications (Li et al., 2013), and pharmacological properties such as anti-cancer, antibacterial and antiviral effects (Jiang et al., 2019). Investigation on *S. miltiorrhiza* showed that its lipophilic fraction possesses marked inhibitory activity against both the proteases (3CL^Pro^ and PL^Pro^) of SARS-CoV, and also found the inhibitory effects of dihydrotanshinone against viral entry in MERS-CoV (Park et al., 2012). In fact, dihydrotanshinone may play a dual role by blocking the entry of coronavirus as well as its post-attachment replication inside the host cell (Kim et al., 2018). Obviously, further validation and clinical trials are needed to establish its antiviral efficacy against SARS-CoV-3CL^Pro^.

## Conclusion

**FIGURE 7:**
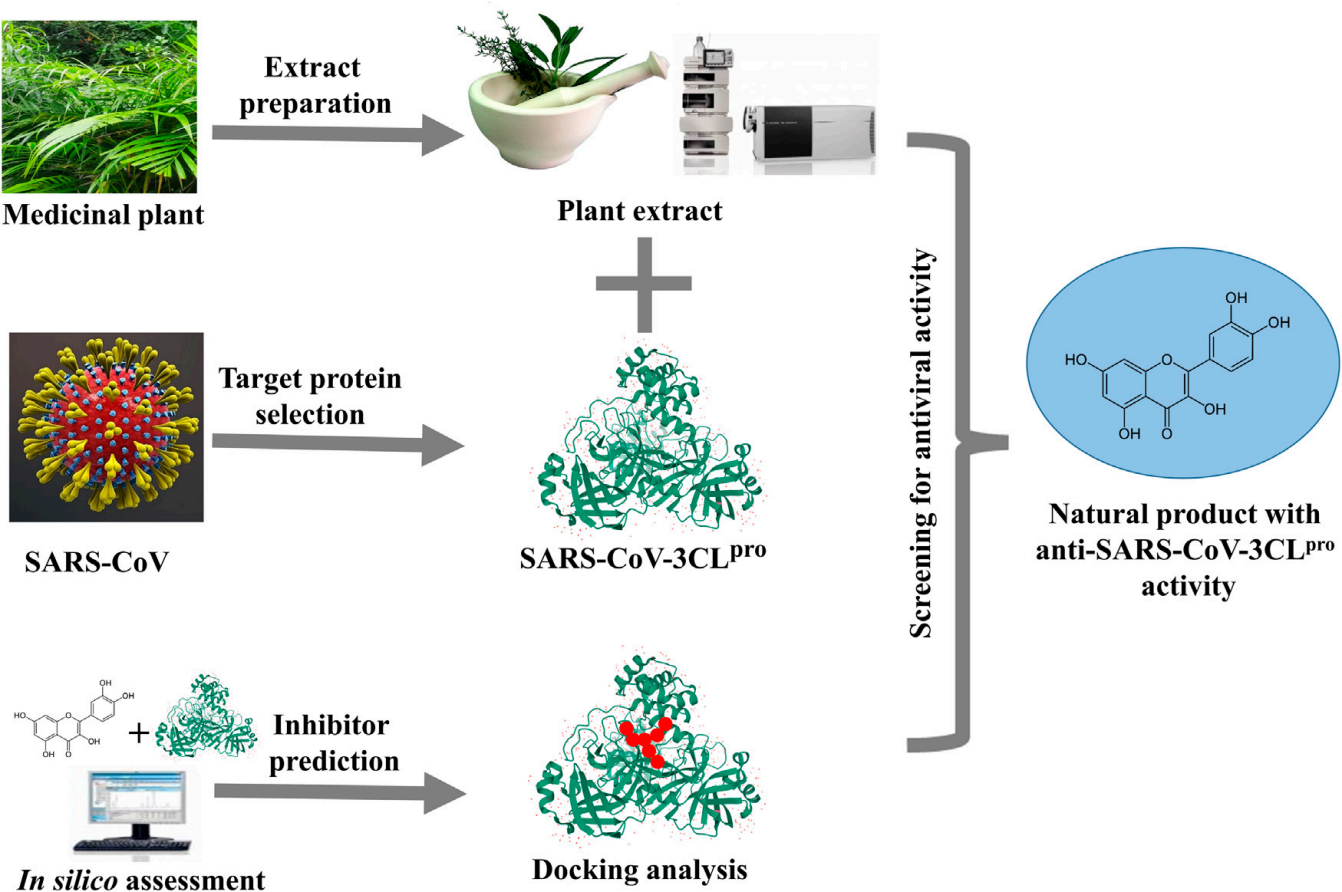
Graphical abstract of the article.

Presently, the world has witnessed the extraordinary outbreak of COVID-19 caused by SARS-CoV-2, raising widespread health concerns. Earlier, in 2003, soon after the emergence of SARS-CoV, the X-ray crystallographic structure of SARS-CoV-3CL^pro^ dimer with a covalently bound inhibitor was elucidated. Since then, numerous inhibitors of 3CL^pro^ enzyme, some of these originating from plants, have been identified, although none of them has reached the clinic till this day. In this perspective, we have discussed several traditional medicinal plant-products screened against 3CL^pro^ activity through *in vitro* and *in silico* studies (Figure 7). In addition, we have proposed promising plant-products which have not yet been explored for inhibition of 3CL^pro^. Needless to say that all the prospective molecules would require *in vivo* antiviral assessment and pharmacokinetic evaluation before going for clinical trial. The structural similarity of HIV- and HCV- protease with SARS-CoV-3CL^pro^ is a promising approach to find useful plant products for COVID-19 therapy, as elaborated in this article. Also, the plants known for treatment of respiratory disorders have been suggested in this regard. Thus, it is a challenge to repurpose and develop these natural products into potent, low molecular weight inhibitors of SARS-CoV-3CL^pro^, with minimum toxicity, to combat the onslaught of emerging coronavirus diseases. We hope that this review will be useful for phytochemists and virologists targeting 3CL^pro^ to identify novel therapeutics against SARS-CoVs.
